# Enhancing agriculture through real-time grape leaf disease classification via an edge device with a lightweight CNN architecture and Grad-CAM

**DOI:** 10.1038/s41598-024-66989-9

**Published:** 2024-07-11

**Authors:** Md. Jawadul Karim, Md. Omaer Faruq Goni, Md. Nahiduzzaman, Mominul Ahsan, Julfikar Haider, Marcin Kowalski

**Affiliations:** 1 Department of Electrical & Computer Engineering, Rajshahi University of Engineering & Technology, Rajshahi, 6204 Bangladesh; 2 Department of Computer Science, University of York, Deramore Lane, Heslington, York YO10 5GH UK; 3 Department of Engineering, Manchester Metropolitan University, Chester Street, Manchester, M1 5GD UK; 4 Institute of Optoelectronics, Military University of Technology, Gen. S. Kaliskiego 2, 00-908 Warsaw, Poland

**Keywords:** Grape leaf disease, Image processing, Lightweight CNN, Embedded system, Grad-CAM, Computational models, Bioinformatics

## Abstract

Crop diseases can significantly affect various aspects of crop cultivation, including crop yield, quality, production costs, and crop loss. The utilization of modern technologies such as image analysis via machine learning techniques enables early and precise detection of crop diseases, hence empowering farmers to effectively manage and avoid the occurrence of crop diseases. The proposed methodology involves the use of modified MobileNetV3Large model deployed on edge device for real-time monitoring of grape leaf disease while reducing computational memory demands and ensuring satisfactory classification performance. To enhance applicability of MobileNetV3Large, custom layers consisting of two dense layers were added, each followed by a dropout layer, helped mitigate overfitting and ensured that the model remains efficient. Comparisons among other models showed that the proposed model outperformed those with an average train and test accuracy of 99.66% and 99.42%, with a precision, recall, and F1 score of approximately 99.42%. The model was deployed on an edge device (Nvidia Jetson Nano) using a custom developed GUI app and predicted from both saved and real-time data with high confidence values. Grad-CAM visualization was used to identify and represent image areas that affect the convolutional neural network (CNN) classification decision-making process with high accuracy. This research contributes to the development of plant disease classification technologies for edge devices, which have the potential to enhance the ability of autonomous farming for farmers, agronomists, and researchers to monitor and mitigate plant diseases efficiently and effectively, with a positive impact on global food security.

## Introduction

Plants have crucial functions in supporting all living organisms on the planet. Given the significance of plants in supporting human survival, it is essential to exercise prudence and implement rigorous protocols when engaging in the detection and study of plant diseases. To prevent the progression of the disease to a severe level, it is imperative to employ appropriate insecticides and genetic modification techniques on crops^1^. Nonetheless, the delayed identification of viral, parasitic, or plague-induced plant diseases necessitates increased use of pesticides on the afflicted plant, hence diminishing the overall crop quality and rate^2^. Accurate classification of plant diseases using artificial intelligence (AI) have multiple benefits in the agricultural industry. First, it improves the overall quality of agricultural goods. Additionally, this approach enables the reduction of excessive use of chemical sprays, such as fungicides and herbicides, contributing to sustainable agriculture. Considering that plants serve as the primary source of sustenance for all living organisms, including humans, it is crucial to conduct further investigations to progress in this domain.

The convolutional neural network (CNN) has become a popular technique in the field of deep learning (DL) and has attracted considerable interest across other scientific domains, especially in the field of agriculture^3^. CNNs have exhibited exceptional efficacy in extracting significant information from images, making them ideal for the assessment of plant health^4^. It possesses the potential to learn complex patterns and subtle variations in leaf textures, colors, and shapes by being trained on large datasets that include both diseased and healthy plants^5^.

The use of embedded edge computing devices improves the effectiveness of this system and enables real-time evaluation of plant health on site^6^. The incorporation of this technology into self-governing rovers/drones represents a significant advancement in the domain of precision agriculture^7^. When paired with advanced disease detection technology, these rovers/drones exhibit exceptional accuracy in navigating vineyards and orchards^8^. These entities have the ability to collect high-resolution imagery and conduct real-time disease evaluations and mapping. This phenomenon not only leads to a reduction in the requirement for manual labor but also facilitates the timely identification and management of plant diseases, hence fostering enhanced crop well-being and overall heightened productivity. Nvidia Jetson Nano, a tiny and low-cost-effective edge device, has been utilized as a tool for disease classification owing to its notable efficiency and computing ability.

The main goal of this study is to create a lightweight CNN architecture from MobileNetV3Large and to engineer it for use on edge devices to create a real-time effective disease classification, categorization and visualization system for grape plants.

This research is supported by the following major novel contributions.

1. Efficient Disease Classification: The main objective is to develop a system for quickly and accurately identifying diseases in grape leaves so that timely interventions are possible. By modifying MobileNetV3Large further, a lightweight CNN model is proposed to process images efficiently while maintaining high classification accuracy. Utilization of the h-swish activation function and squeeze-and-excitation structures has also been proposed to efficient extract important features associated with diseases in grape leaves.
2. Deployment on Edge Devices: A CNN-based disease detection model was seamlessly incorporated into an edge device. These gadgets will be fixed to self-driving rovers/drones, enabling in-field real-time image capture and analysis. This deployment strategy ensures quick disease assessment with low latency since all the test are run locally. Also it comes with an advantage of bandwidth conservation and offline capability as there is no need for internet or cloud processing.
3. Real-Time Explainable AI (XAI) Representation with Grad-CAM: Unlike most of the research works that utilized Grad-CAM or any other heatmap visualization model for only testing, this research focus on implementing it for real time operation for precise targeting treatment measures, like selective pruning or targeted pesticide application, by highlighting regions of interest linked to particular diseases.

## Literature review

Several authors have implemented different CNN architectures for grape leaf disease classification in the last decade while most of these studies focused on developing models based on large architectures for classification. However, recent studies are beginning to focus more on lightweight-based models for classification. A literature review of each of these works is provided, starting with models consisting of large architectures requiring high computational requirements and followed by lightweight models developed for running on edge devices.

Transfer learning has been used in a number of studies with large CNN architectures, which are computationally demanding despite their effectiveness. For instance, Paymode et al.^9^ classified grape and tomato leaf diseases using VGG16, and they did so with a high accuracy of 98.40%. In another similar work, AlexNet, VGG-19, and Inception v3 were evaluated by Morellos et al.^10^, with Inception v3 attaining up to 100% validation accuracy. AlexNet was also utilized by Aravind et al.^11^, who increased its accuracy to 99.23% by utilizing a multiclass support vector machine. With the help of Nagaraju et al.^12^, VGG-16 was improved to classify apple and grape leaf diseases with a 97.87% accuracy rate. A new method for detecting grape leaf diseases called the UnitedModel was introduced by Ji et al.^13^. This method utilizes a combined convolutional neural network (CNN) architecture that incorporates the advantages of both GoogLeNet and ResNet. The purpose of this model is to distinguish between healthy leaves and leaves damaged by common grape diseases, such as black rot, esca, and isariopsis leaf spot. The UnitedModel had exceptional performance on the PlantVillage dataset, with an average validation accuracy of 99.17% and a test accuracy of 98.57%. Liu et al.^14^ proposed a novel CNN-based model, the DICNN, from scratch. The original dataset was collected from the field and public dataset repository and was used to generate a total of 107,366 grape leaf images of 7 classes. Upon accuracy comparison with other models such as GoogleNet and ResNet34, the proposed model (97.22%) generated increased accuracies of 2.97% and 2.55%, respectively. Atila et al.^15^ used the EfficientNet architecture, specifically emphasizing the B4 and B5 models, for the purpose of categorizing plant leaf diseases. The dataset included 55,448 original and 61,486 enhanced photos, covering 39 categories of 14 distinct plant species. The B5 model achieved an impressive accuracy of 99.91% on the original dataset, while the B4 model achieved an even higher accuracy of 99.97% on the enhanced dataset. Shovon et al.^16^ proposed an ensemble learning method for the classification of rice and beetle leaf disease with the IRV2_EV2L_Xcep model. As suggested by Nagasubramanian et al.^17^, a 3D-CNN model was used to extract characteristics from 3D hyperspectral data that span both the spatial and spectral dimensions with a classification accuracy of 95.57%, and an F1 score of 87% was obtained. Even after having high accuracies, these models are not suitable for real-time applications on edge devices due to their large number of parameters and high computational demands. Also many of them^9–12^ lacked other performance metrics such as precision, recall, and F1-score, which are also important for checking the robustness of the model.

The focus of recent research has shifted to lightweight models that can be deployed on edge devices and operate at high accuracy. For a variety of plant leaves, Anari et al.^18^ developed a lightweight ResNet-18 model that achieved high accuracies but lacked practical validation on edge devices. This method produced better results, but it required more computer power. Rao et al.^19^ used AlexNet as the transfer learning model and stochastic gradient descent (SGD) as the optimizer to achieve an accuracy of 98.85% for grape leaves and 90% for mango leaves. Tang et al.^20^ applied the SE module within the ShuffleNet network and proposed a lightweight convolutional neural network. The network's performance was assessed using publicly available data on four different grape diseases, and the training set's accuracy was 99.14%. In another work, plant disease detection using MobileNet architectures was enhanced by Parez et al.^21^ and Chen et al.^22^. On the PlantVillage dataset, Chen et al.'s MobileNet-Beta achieved remarkably high accuracy. But the evaluation of these studies' practical applicability is limited by the absence of comprehensive performance metrics like precision, F1-score, or AUC. Novel models that integrate Inception and SE-blocks, as well as efficient channel attention techniques, were presented by Xie et al.^23^ and Wang et al.^24^. Peyal et al.^25^ used a lightweight 2D CNN model covering 12 infected and 2 healthy states to classify tomatoes and cotton crops into 14 categories. The model, which outperforms pretrained models with 97.36% average accuracy was incorporated into the "Plant Disease Classifier" Android app. Also, it provided insights into disease identification by utilizing Grad-CAM to provide visual explanations.

These models are claimed to be appropriate for real-world agricultural applications because they showed good detection accuracy. However, their effectiveness was only tested on particular datasets, which begs the question of how reliable they are in various environments. The works discussed here, as presented in papers^9–12,14,18,21,22^, showed a common aim of achieving high accuracy by leveraging complex architectures or ensemble methods. Although the missing results on real-time validation of these models on real-world datasets and edge devices leave a significant gap in their studies. Most of these works suffer from overfitting by using limited and unbalanced datasets. Also, the absence of comprehensive performance metrics further limits the evaluation of model robustness. In order to cover these gaps, the following research focuses on developing an accurate and lightweight model, for grape leaf disease classification that can be deployed on edge devices and has been validated on real-world tests.

## Materials and methods

### Overall architecture of the proposed system

**Figure 1:**
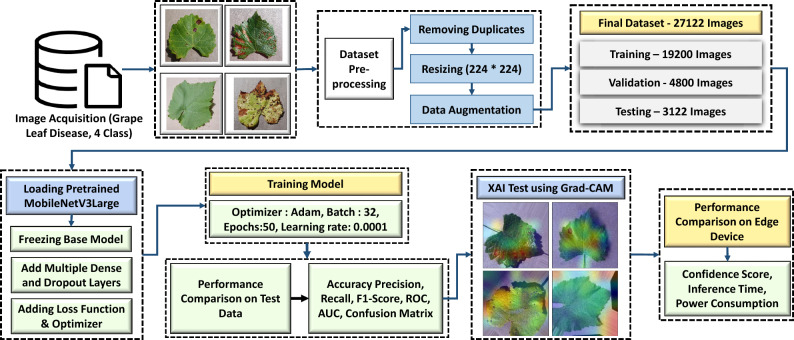
Block diagram of the proposed research framework.

For this research, a preplanned approach was developed for efficient and rapid development of the model and its testing. The complete process from data collection to preprocessing, from customizing the proposed model and training it to evaluating the test data, is explained in detail and shown according to the block diagram in Fig. 1.

### Image acquisition

**Figure 2:**
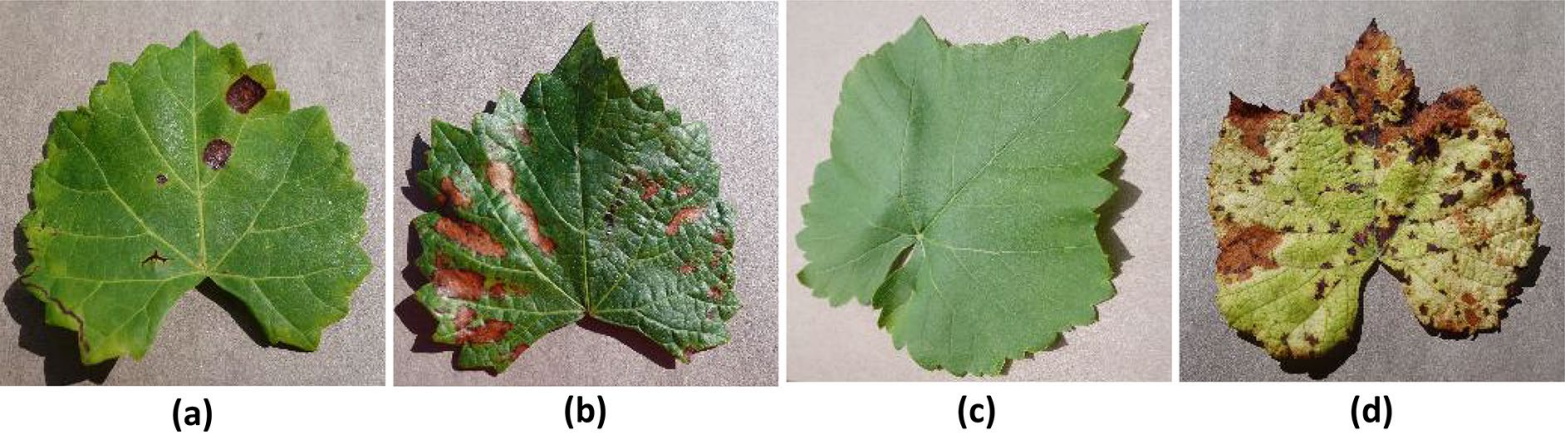
Four classes of grape leaves from the dataset. (**a**) Black rot, (**b**) ESCA, (**c**) healthy, (**d**) leaf blight.

The dataset for this research was obtained from the “Grapevine Disease Dataset (Original)”^26^, which contains four classes with a total of 7222 images for the training part and 1805 images for the test part (Fig. 2). Each class contains approximately 1600–1900 training images with 400–450 test images (Table 1). The images were originally unbalanced RGB images of 256 × 256 each in size.

**Table 1:** Original downloaded dataset with distribution of grape leaf classes for training and testing.

Class type	Train data	Test data
Black rot	1888	472
ESCA	1920	480
Healthy	1692	423
Leaf blight	1722	430

The dataset^26^ for grape disease obtained from Kaggle consisted of a small amount of data, which is not suitable for proper model building. When a model is equipped with a large number of parameters but is given only a limited amount of data, its ability to effectively learn the underlying patterns is compromised, leading to vulnerability to overfitting^27^. In addition to the presence of imbalanced data within each class, it is important to acknowledge that such data can lead to the development of a biased model.

### Preprocessing of image data

One of the most important steps in obtaining data ready for CNN model training is data preprocessing. Upon inspecting the dataset, there were duplicate/redundant images in every class, which resulted in increased training time and memory consumption. Additionally, few images were misclassified as the wrong class by the provider, which, upon initial training, resulted in poor accuracy and incorrect predictions. Therefore, duplicate and misclassified images were manually removed.

**Figure 3:**
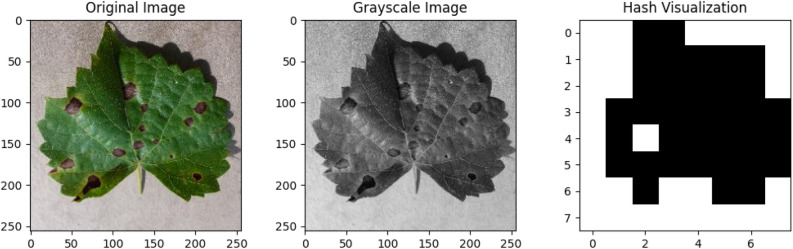
Image hash technique steps (average hash) from left to right.

To remove duplicate images, an image hash algorithm (average hash) was used. This procedure normalized every image from the dataset to a consistent, compact dimension, usually an 8 × 8 pixel grid. A binary hash was created after each image that is transformed to grayscale and resized to a fixed size. The mean pixel intensity was calculated, and the image was then resized to its original size. Every bit in the hash encodes information about a pixel's intensity greater or less than the mean value^28^. Figure 3 presents an original image and its corresponding hashed image. When two or more hashes were found to be the same, the duplicate image was found and removed permanently.

**Figure 4:**
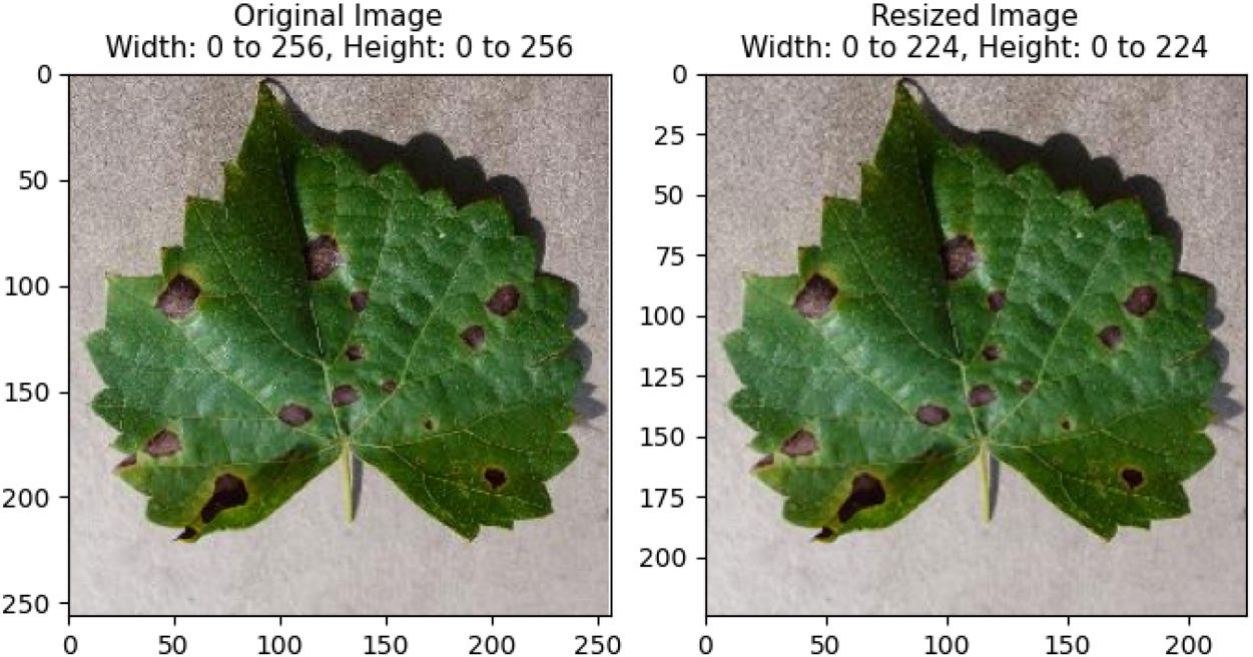
Resizing images from 256 × 256 to 224 × 224 pixels.

In addition to working with edge devices and lightweight models, the usual image sizes needed to be reduced. Most of the lightweight models support 224 × 224 pixels. The original dataset downloaded from Kaggle had an image dimension of 256 × 256, which was converted to 224 × 224 using the Pillow Library, The images were subjected to a conversion process that transformed them into RGB color mode, thereby guaranteeing a comprehensive representation of colors. Resizing an image was accomplished by utilizing the default antialiasing resampling filter. This filter effectively preserved the quality of the image while simultaneously reducing the occurrence of aliasing artifacts. A comparison between a normal image and a resized image is shown in Fig. 4.

**Figure 5:**
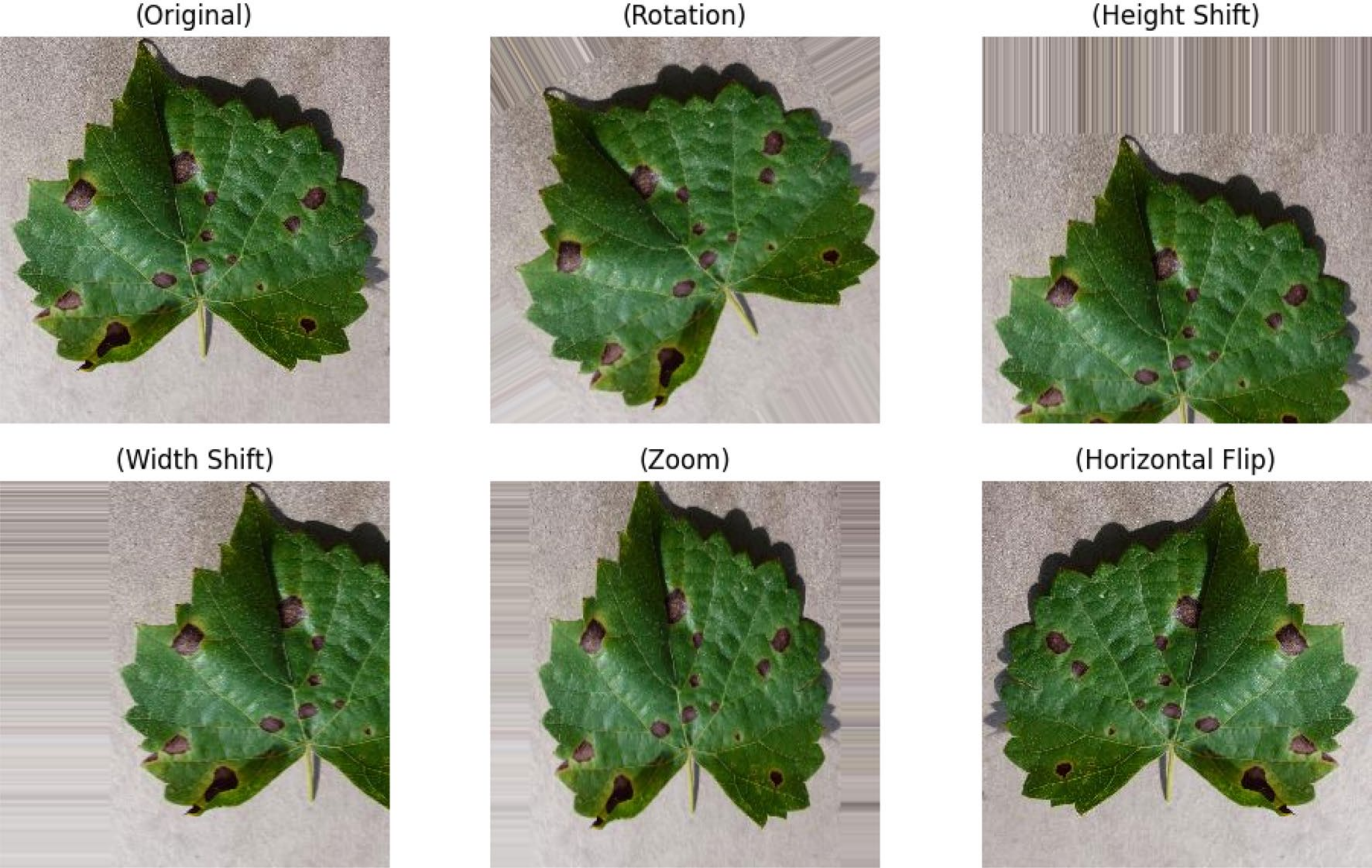
Data augmentation to generate additional images from a single image.

An unbalanced dataset can lead to a number of problems during the process of training machine learning models, including biased model learning, poor generalization, imbalanced loss functions, and misleading evaluation matrices. Dataset balancing was implemented using image augmentation techniques in which 5 new augmented images were created from each image using width shift, height shift, rotation, flip, and zoom augmentation techniques. This approach contributed to improve the model’s performance in three primary ways: increasing the size of the dataset without requiring the collection of additional data, improving model generalization by exposing the dataset to a diverse range of data variations and enhancing model robustness to real-world variations such as lighting and orientation. Finally, for each class of the training dataset, 6000 images were obtained after removing duplicate images. Similar method was also applied to increase the size of the test dataset. It was ensured that under no circumstances, the training and test data had similar images. Figure 5 shows examples of image augmentation applied to create additional images, and Table 2 presents the final distribution of images in the dataset after augmentation.

**Table 2:** Final dataset after augmentation.

Class type	Train data	Validation data	Test data
Black rot	4800	1200	780
ESCA	4800	1200	781
Healthy	4800	1200	780
Leaf blight	4800	1200	781

### Customizing pretrained CNN model

The CNNs have considerably revolutionized the fields of agriculture and image processing with their remarkable capability to accurately process through the visual input. Today, thanks to deep learning frameworks and vast labeled image datasets, it has become one of the go-to choices for a variety of image-related tasks. In the field of image classification, many networks like VGG16, ResNet, Inception have shown exceptional performance as state-of-the-art (SOTA) approaches by using transfer learning. However, portability constraints encourage the development of lightweight CNN models of multiple layers that can capture rich image features and patterns.

**Figure 6:**
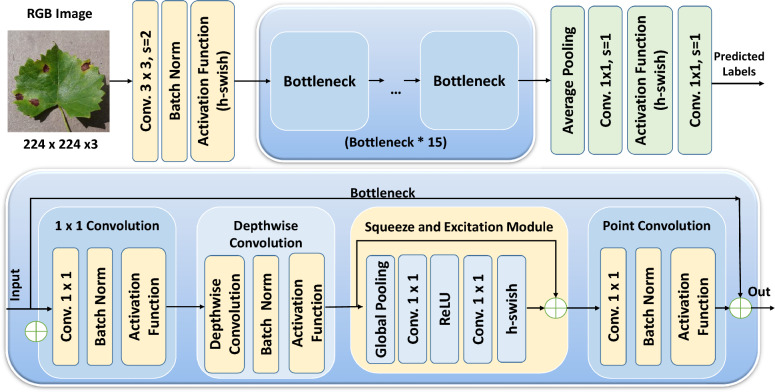
MobileNetv3Large architecture.

Scalability and the resource constraint in different situations such as for a mobile device, edge computing platform, and any real-time application are the crucial concerns. Furthermore, energy is a major concern and we need to conserve battery life for longer reasons in portable devices and it is also essential to reduce the energy consumption especially in edge computing scenarios. Lightweight models largely help address these concerns by minimizing computational burden. We choose MobileNetV3Large as the model of interest for transfer learning, and this is the third version of the MobileNet class of models. To keep its design lightweight, MobileNetV3Large employs multiple techniques, including inverted residuals, expanded activation functions, network architecture search, squeeze-and-excitation blocks, and a light classification head^29^. This characteristic renders it a very suitable option for integration into embedded systems, including Internet of Things (IoT) devices and mobile devices, which often possess constrained computational capabilities. Figure 6 shows the model architecture for Mobilenetv3 with its individual blocks.

The bottleneck component of MobileNetV3Large which enhanced input feature space with the help of 1 × 1 convolution. Then, depthwise convolutions were applied with different kernel sizes. There is also an optional integration of a squeeze-and-excitation (SE) layer. Finally, the feature space was reduced back to the size of the original image with a pointwise convolution. Alternatively, the ResNet architecture includes a skip connection as a ResNet style skip connection. This skip connection is implemented when the shape of the input tensor is equal to the shape of the output tensor so as to increase the architectural robustness. This operation, called a convolutions, served to increase the depth of the feature map, thus increasing the capacity of the network to describe data. The expansion ratio, commonly represented as the 'expand ratio', is a hyperparameter that controls the magnitude of the expansion. The 1 × 1 convolution layer provides the ability to exert flexible control over the quantity of output channels that are generated. The process of expansion plays a crucial role in the architecture of MobileNetV3Large, as it prepares the feature map for later activities.

**Figure 7:**
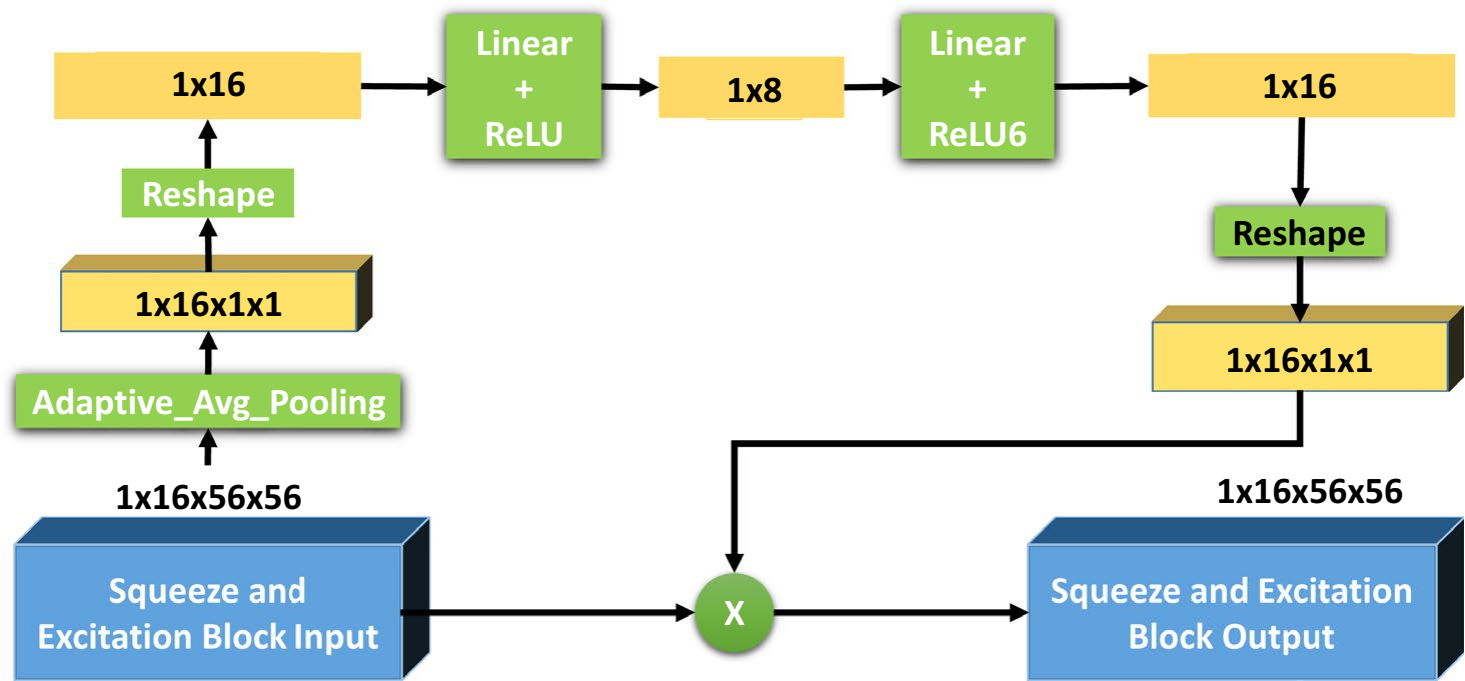
Detailed block diagram of the squeeze and excitation block of MobileNetV3Large.

Using SE layer (Fig. 7) required the dependency between the channels to be used and information from the feature map, which was dynamically calibrated. The feature maps from the previous convolutional layer were input into the SE block. The image was transformed in 3D tensor with dimensions such as 1 × 16 × 56 × 56. Here, 16 stands for the number of channels and 56 × 56 is the spatial dimensions of feature maps. The first operation in the SE block, the adaptive average pooling took spatial dimensions (56 × 56) of each channel and compresses into a single number. As a result, a tensor of size 1 × 16 × 1 × 1 was obtained, which condenses the overall spatial information of each channel into a single value. This stage aggregated the feature responses throughout the spatial domain, providing a comprehensive representation of the input characteristics on a global scale. The basic effect of reshape was to keep the content of the data the same as the input data and change the size so that it can be adapted to subsequent fully connected FC layers. After this, the overall information in the tensor was still there, except it was arranged in a 1 dimensional vector with 16 elements that had a fully linked layer whose dimensionality had been reduced to 8 fed into the transformed tensor. This was a bottleneck layer, capable of picking up the most prevailing aspects of the channels. Then a Rectified Linear Unit (ReLU) was used as activation function, which introduced nonlinearity and allowed for the learning of complex functions. Another fully linked layer was used to restore the dimensionality of the features to their original number of channels, for instance, from 8 to 16. Then an activation function ReLU6 was use which was similar to ReLU but it limits the top value to 6 to prevent over-activation.2. We reconverted the output of the fully connected (FC) layers into a three-dimensional tensor with only one spatial dimension (1 × 16 × 1 × 1). The values in this tensor were the learned weight that will be used to recalibrate the original feature maps. These adjusted weights were then multiplied with the original feature maps matching the calculation channel dimension-wise. Therefore, each single channel of the original input was multiplied by the scalar relating to the same channel of the recalibrated tensor. This process rescaled the feature maps, in order to enhance significant elements and suppress less important features. The result of the scaling operation was the output SE output, a tensor with the same dimensions as the SE input (1 × 16 × 56 × 56). As result of giving weights based on their importance decided by the SE block, the recalibrated feature maps now were a more refined set of features to extract. These processes were specifically designed to allow the network to learn which features to boost and which to suppress, allowing the optimization of network performance over a variety of tasks that rely on feature discrimination.

MobileNetV3Large has been specifically designed for CPUs by utilizing a combination of hardware-aware network architecture search (NAS) to enhance both the structure and selection of nonlinear functions. Neural architecture search automated the process of determining optimal layer configurations and connection topologies by evaluating their performance on a validation dataset^30^. Additionally, the proposed model replaced the ReLU nonlinearity used in earlier versions with the Hard Swish activation function, which approximated the Swish function using piecewise linear segments.

**Figure 8:**
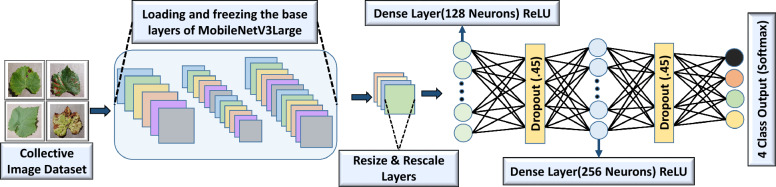
Loading of pre-trained model (MobileNetV3Large) and addition of layers to construct the proposed network.

In this study, six models were trained with different types of lightweight CNN architectures and deployed on an edge device to identify the best performing one based on changes in accuracy, visualization, inference speed (CPU), power consumption and confidence. The final selected models were NASNetMobile, MobileNetV3Large, MobileNetV3Small, DenseNet121, EfficientNetV2B1 and EfficientNetV2B2 in their customized forms. Figure 8 shows the relevant diagram of the customized model built upon the pretrained weights of MobileNetV3Large. After loading the image data, all the images were shuffled to reduce overfitting during training. They were then preprocessed using the corresponding model’s built-in preprocessing functions. Finally, the pretrained models were loaded with max pooling, and the top layers were removed because we used our own fully connected layers in this part. Subsequent weight parameters were initialized using pretrained values that were primed on the ImageNet dataset. On average, each synset was represented by approximately 1000 images sourced from ImageNet, which provides a substantial collection of meticulously labeled and organized images (10 million) for the majority of concepts within the WordNet hierarchy^30^. All the pretrained layers of each model were frozen because they were fully utilized, allowing the models to run more efficiently during training. Once the input layer of the pretrained model was replaced, the custom layers underwent resizing and normalization. The fine-tuning process thus consisted of stacking a number of layers on top of the output of the pretrained model. First layer, a fully connected, dense layer with 128 neurons used by nonlinearity by ReLU activation. To combat the overfitting issue, a dropout layer with a dropout rate of 0.45 was added. During training, we did this by turning off some of the input units at random, which we zeroed out. Appropriately, the process was repeated with another dense layer of 256 neurons, followed by, once more, a dropping layer that uses the same dropping rate. The last layer in the series consisted of a dense layer with 4 neurons, with a softmax activation function used to represent the four classes. Using the softmax activation, the output values were turned into a probability distribution (ensuring normalization), when we have four classes. The loss function, “categorical_crossentropy”, was optimized using Adam with a learning rate equal to 0.0001. Overall, Adam is already well known to be effective and robust for optimizing complex, high dimensional models^31^. The execution of the model during training was quantified by this, since it was assigned, hence serving as the objective to be optimized by the optimizer for it to be minimized. The model will thus make high-confidence predictions about inputs of the correct class by penalizing the incorrect predictions more heavily^32^.

The end result is a personalized model that integrates the feature extraction skills of the pretrained model with supplementary layers designed for a particular classification job, showcasing a prevalent technique in transfer learning. Consequently, this process facilitates the development of deep learning models that are both efficient and tailored to specific tasks^33^.

The following hyperparameters (Table 3) were kept the same while training all six models. The learning rate was kept low to avoid overfitting and poor generalization of the models. Since obtaining a higher accuracy of the model was the first goal, monitoring metrics were set to observe the training accuracy only and save it when the latest epoch reached a higher accuracy than the last epoch.

**Table 3:** Hyperparameter values for training the model.

Parameter name	Applied value
Batch count	32
Image size	224 × 224
Image type	RGB
Dropout	0.45
Activation function before output layer	ReLU
Activation function at output layer	Softmax
Monitoring metrics	‘accuracy’
Optimizer	Adam
Learning rate	0.0001
Loss function	Categorical crossentropy
Number of iterations	50

**Figure 9:**
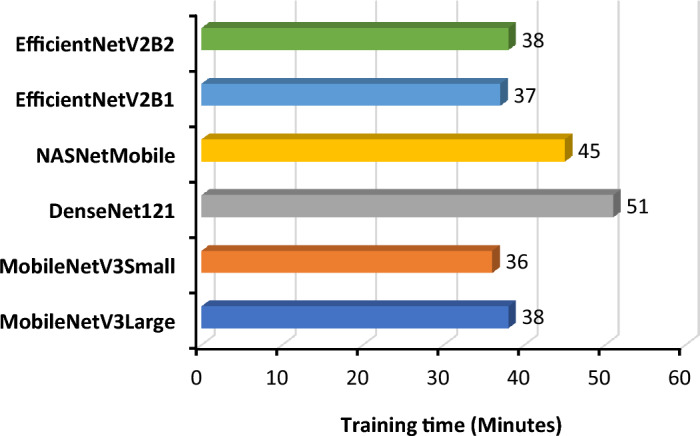
Training time for each of the models with the same parameter.

Model training, particularly for deep learning techniques, involves computationally intensive tasks such as matrix multiplication and gradient calculations. For quicker training, the models were trained on the Kaggle platform, where an Nvidia Tesla P100 GPU with 16 GB of memory was the key hardware. The system achieved a peak performance of 9.3 teraflops in double-precision calculations, which is a vital characteristic for executing model training. A comparison of the training times for different models is shown in Fig. 9.

### Grad-CAM visualization

The Gradient-weighted Class Activation Mapping (Grad-CAM) technique shows salient insight about why the DL models made a prediction visualizing the more relevant regions of the input image influencing the decision process. The process commences with the state-of-the-art deep neural network operation (forward pass) to produce raw prediction scores for every class as it was based on the incoming image^34^. The initial scores are the biggest part, setting the stage for the other steps. In the next stage, backpropagation was started after forwarding pass was done. During the process of backpropagation, the gradients of the predicted class scores were calculated with respect to the feature maps originating from the final convolutional layer. The depicted gradients served as indicators of the model's responsiveness to alterations in the feature maps. The significance-weighted gradients are frequently subjected to spatial averaging, leading to weighted aggregation that encompasses the essential regions accountable for the classification of the network^35^.

**Figure 10:**
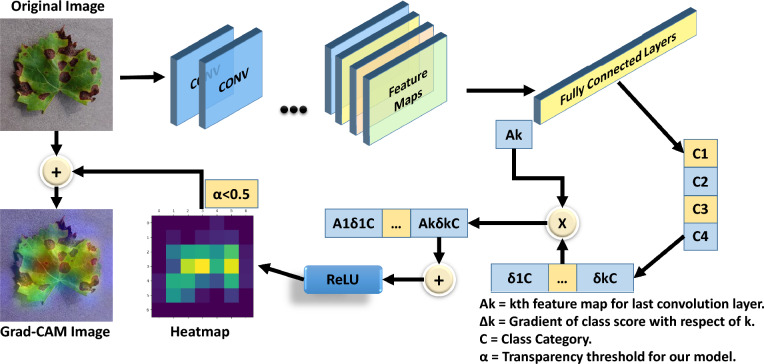
Grad-CAM architecture with transparency thresholding.

For this research, Grad-CAM was implemented on bulk images from the test dataset as well as on single real-time leaf images. The goal was to differentiate diseased leaves from healthy leaves by highlighting them. The general Grad-CAM visualization technique (Fig. 10) was slightly modified in this work so that only diseased classes are marked while avoiding healthy classes, as they are not important for real-time visualization.

The procedure started with the importation of essential libraries for numerical computations, picture manipulation, and deep learning capabilities.

It then imports the customized MobileNetV3Large model from a specified file directory. Subsequently, essential parameters such as picture dimensions and the last convolutional layer of the model were selected, which was necessary for the Grad-CAM approach. Finally, a picture was imported, scaled, and preprocessed to conform to the input specifications of the model.

The process prioritized the feature maps obtained from the final convolutional layer by using the gradients of a selected target class from the network's output to allocate significance weights to each feature map. This process entailed generating a specific model that produces the output of the last convolutional layer (Conv_1 for MobileNetv3_Large) and the final predictions. The code used TensorFlow's gradient computation to identify the regions in the picture that have the most impact on the model's prediction. These feature maps were merged using the weights, and a rectified linear unit (ReLU) function was used to retain only the features that had a favorable impact on the classification prediction. The gradients were computed and subsequently averaged to construct a heatmap.

The last step involved overlaying this heatmap over the original picture. The heatmap underwent colorization and resizing to align with the proportions of the source picture. The original picture was superimposed with a designated degree of transparency, yielding a composite image that graphically depicts the focal regions for the model's decision-making procedure. However, the transparency level of the heatmap before superimposing was increased to a certain level to focus only on diseased pixels and neglect healthy pixels. Several tests were performed to determine the perfect transparency of the proposed model. The overlay picture offered a clear and easy-to-understand visual representation of the model's predictions of diseased areas only. This implementation serves as a tangible demonstration of improving the comprehensibility of deep learning models, facilitating a better understanding and reliance on their judgments.

### Computational specification and evaluation metrics for model performance

The model was trained by NVIDIA Tesla P100 GPUs, along with an approximate GPU memory capacity of 16 GB, under the complimentary GPU service extended by Kaggle. The Tesla P100 is outfitted with a collective sum of 3584 CUDA cores, which have been purposefully engineered to execute parallel computations. The customary arrangement of this product generally consists of 16 GB of High Bandwidth Memory (HBM2), which offers a significant memory bandwidth crucial for efficiently handling large datasets and complex neural networks.

To determine the best performance of the trained models, the test dataset was used to determine the evaluation metrics. The accuracy, precision, recall, F1 score, and area under the curve (AUC) were calculated and furthermore, the model’s inference time for both .h5 and .tflite, Giga Floating Point Operations Per Second (GFLOPS) and the power consumption of each model were measured to identify the optimal model for classification in edge devices.

Accuracy calculation involved determining the proportion of accurately recognized leaves, including both infected and healthy leaves, out of the total number of leaves that were examined. Precision quantified the model's ability to accurately forecast the presence of disease in leaves among all the leaves classified as diseased. The diseased leaf prediction accuracy was determined by the ratio of accurately predicted diseased leaves (true positives) to the total number of leaves identified as diseased (the sum of true positives and false positives). A high level of accuracy implies that when the model predicts that a leaf is unhealthy, there is a high probability that the leaf is truly infected. The recall quantified the model's capacity to accurately detect all existing sick leave. The diseased leaf prediction accuracy was determined by the ratio of accurately predicted diseased leaves (true positives) to the total number of leaves that are truly diseased (the sum of true positives and false negatives). A high recall score suggests that the model is proficient at accurately detecting the majority of sick leaves. The power consumed by the CPU of the edge device during prediction was measured using the Jtop library. As the goal was to choose a suitable model for lightweight application, the computational complexity of each of the trained models was measured through GFLOPS measurements.

## Analysis of experimental results

### Comparison among the models

**Figure 11:**
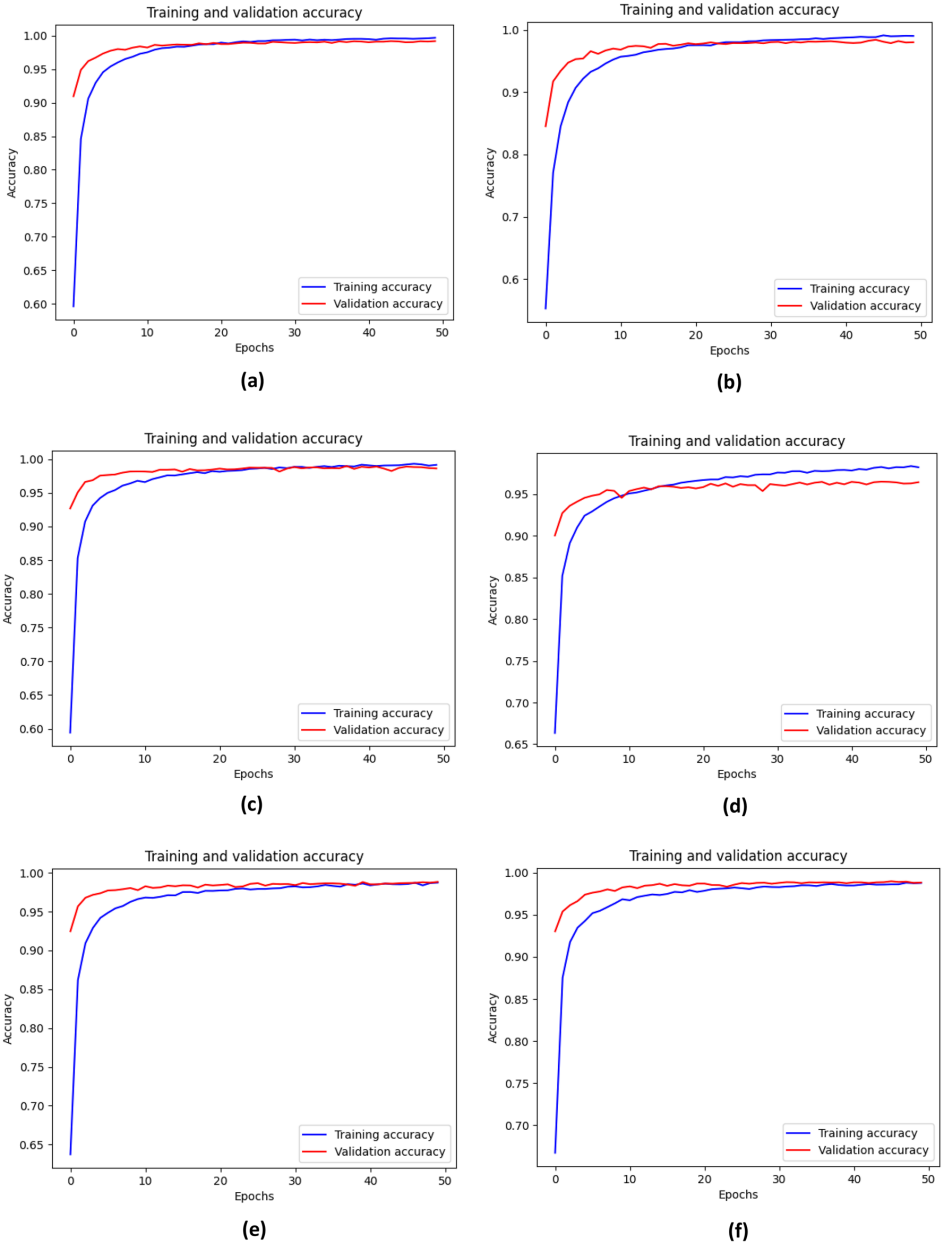
Graph for training and validation accuracy of the models (**a**) MobileNetV3Large, (**b**) MobileNetV3Small, (**c**) DenseNet121, (**d**) NASNetMobile, (**e**) EfficientNetV2B1, and (**f**) EfficientNetV2B2.

**Figure 12:**
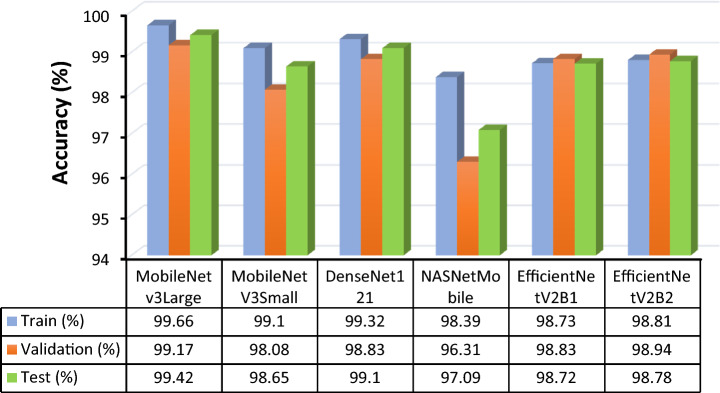
Bar graph and data table of all six models for training, validation and test accuracies.

All six different models were trained with the same metrics and dataset. During the training of these models, the optimizer, training size, batch number, validation size, learning rate, and number of iterations were kept the same. An accuracy comparison of the chart shows that MobileNetV3Large outperformed the other models in terms of training (99.66%), testing (99.42%) and validation accuracy (99.17%). The closest performance to that of MobileNetV3Large was shown by DenseNet121, with 99.1% test accuracy, while NASNetMobile responded with a very poor test accuracy of 97.09%. The accuracy of all the models was stable throughout the training process, which demonstrated that the data contained less noise, avoided gradient overshooting and became stuck at local minima. The following graphs and bar charts in Figs. 11 and 12 show the training and validation accuracies of all six models.

**Figure 13:**
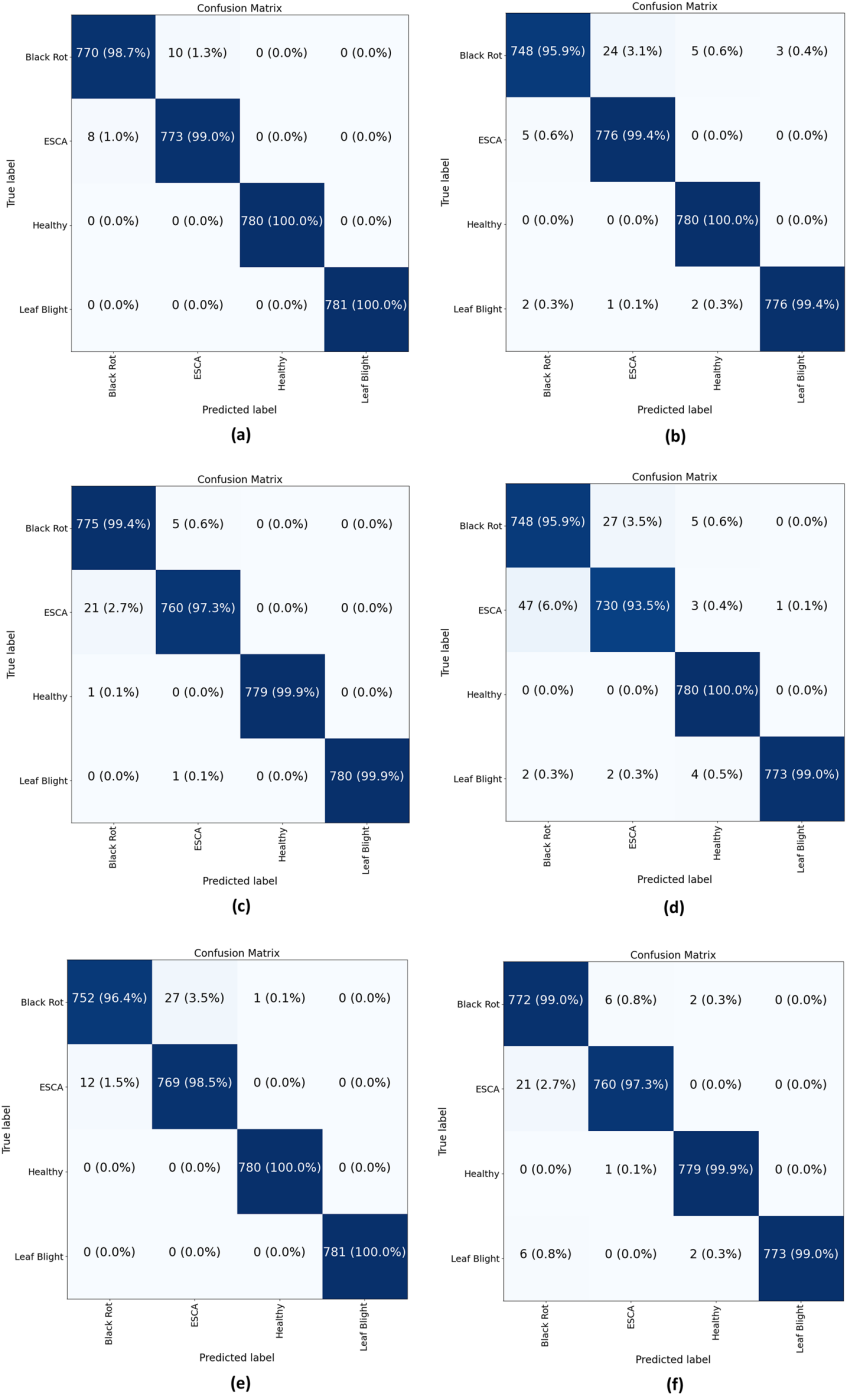
Confusion matrix for disease class from each of the models: (**a**) MobileNetV3Large, (**b**) MobileNetV3Small, (**c**) DenseNet121, (**d**) NASNetMobile, (**e**) EfficientNetV2B1, and (**f**) EfficientNetV2B2.

In the confusion matrix, four distinct classes were established based on the dataset, with one class representing healthy and the remaining classes representing diseased (Fig. 13). A total of 780 authentic photos were allocated to each class. The MobileNetV3Large, DenseNet121, and EfficientNetV2B2 models demonstrated superior performance based on their respective confusion matrices. DenseNt121 and EfficientNetV2B2 demonstrated marginally superior performance compared to MobileNetv3Large in the precise categorization of black rot. MobileNetV3Small had demonstrated improved classification performance for the ESCA class. Overall, MobilenetV3Larges yielded the most favorable results.

To conduct a more comprehensive assessment of the performance of each model and of their respective classes, metrics such as precision, recall, and F1 score were computed. MobileNetV3Large shown the most favorable outcomes for all the classes, achieving precision scores of 98.97%, 98.72%, 100%, and 100% for black rot, ESCA, healthy and leaf blight, respectively. Conversely, NASNetMobile had the lowest performance, particularly for the black rot class (93.85%) and the ESCA class (96.1%). In contrast, the recall results yielded the same output as the precision results. The MobileNetv3Large model achieved a recall of 98.70% for the classification of black rot, whereas the NASNetMobile model achieved a recall of 95.89%. EfficientNetV2B2 had demonstrated comparable performance to that of MobileNetV3Large. When comparing the model accuracy for each class, it is observed that both MobilenetV3Large models exhibited higher F1 scores, which surpass the other models. The performance of NASNetMobile was suboptimal, with a score of 94.16% or less. The corresponding data in Table 4 shows the comparisons between the precision, recall and F1 score of the individual models.

**Table 4:** Precision, recall and F1 score data for each of the six trained models.

Class name	Model	Precision	Recall	F1-score	Model	Precision	Recall	F1-score
Black rot		0.989717	0.987179	0.988447		0.938519	0.958974	0.948637
ESCA		0.987229	0.989757	0.988491		0.961792	0.934699	0.948052
Healthy	MobileNetV3Large	1.000000	1.000000	1.000000	NASNetMobile	0.984848	1.000000	0.992366
Leaf blight		1.000000	1.000000	1.000000		0.998708	0.989757	0.994212
Black rot		0.990728	0.958974	0.974593		0.984293	0.964103	0.974093
ESCA		0.968789	0.993598	0.981037		0.966080	0.984635	0.975269
Healthy	MobileNetV3Small	0.991105	1.000000	0.995533	EfficientNetBV2B1	0.998720	1.000000	0.999359
Leaf blight		0.996149	0.993598	0.994872		1.000000	1.000000	1.000000
Black rot		0.972396	0.993590	0.982879		0.966208	0.989744	0.977834
ESCA		0.992167	0.973111	0.982547		0.990874	0.973111	0.981912
Healthy	DenseNet121	1.000000	0.998718	0.999359	EfficientNetBV2B2	0.994891	0.998718	0.996801
Leaf blight		1.000000	0.998720	0.999359		1.000000	0.989757	0.994852

**Figure 14:**
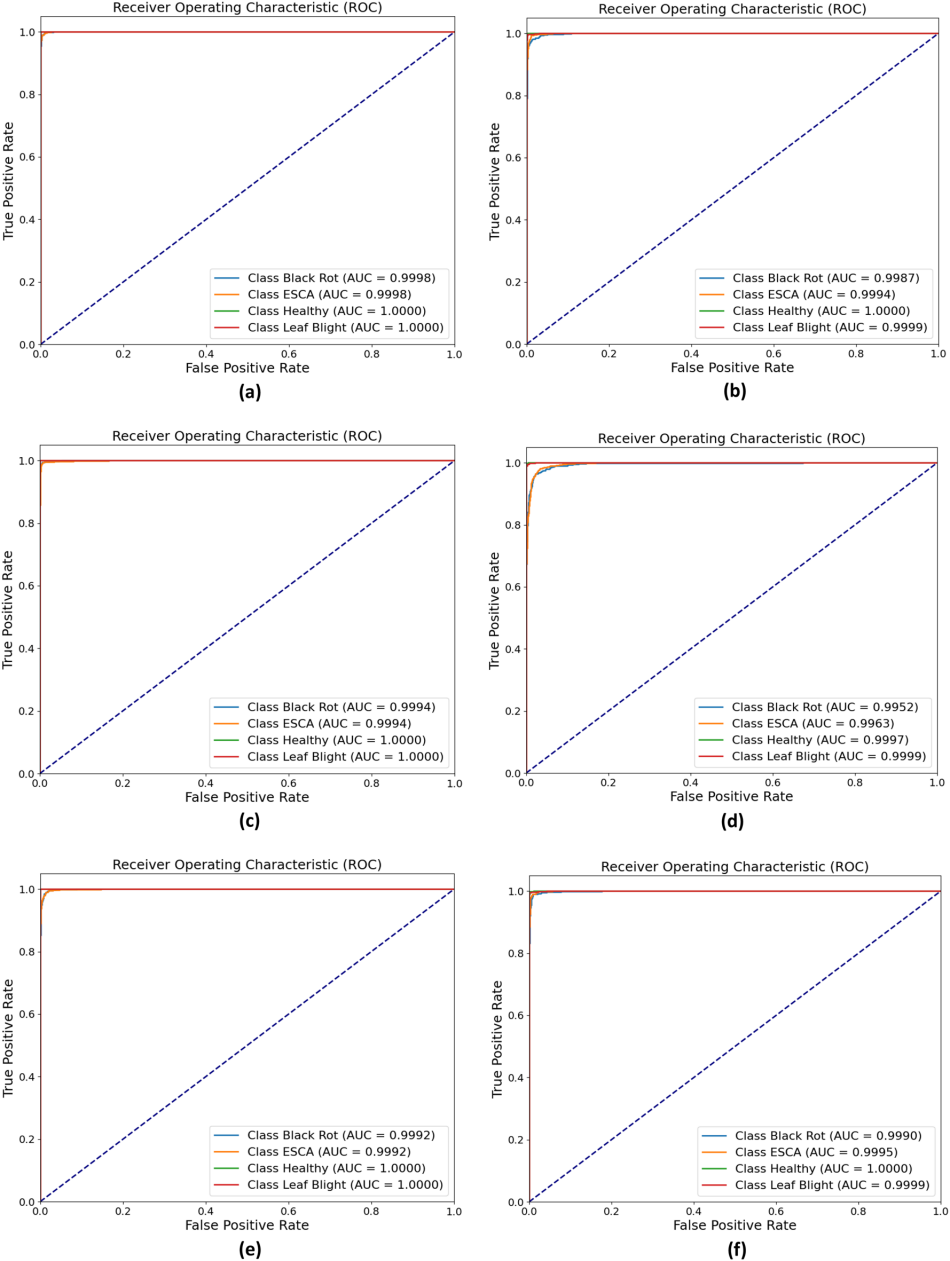
ROC curves for each of the models: (**a**) MobileNetV3Large, (**b**) MobileNetV3Small, (**c**) DenseNet121, (**d**) NASNetMobile, (**d**) EfficientNetV2B1, and (**f**) EfficientNetV2B2.

**Figure 15:**
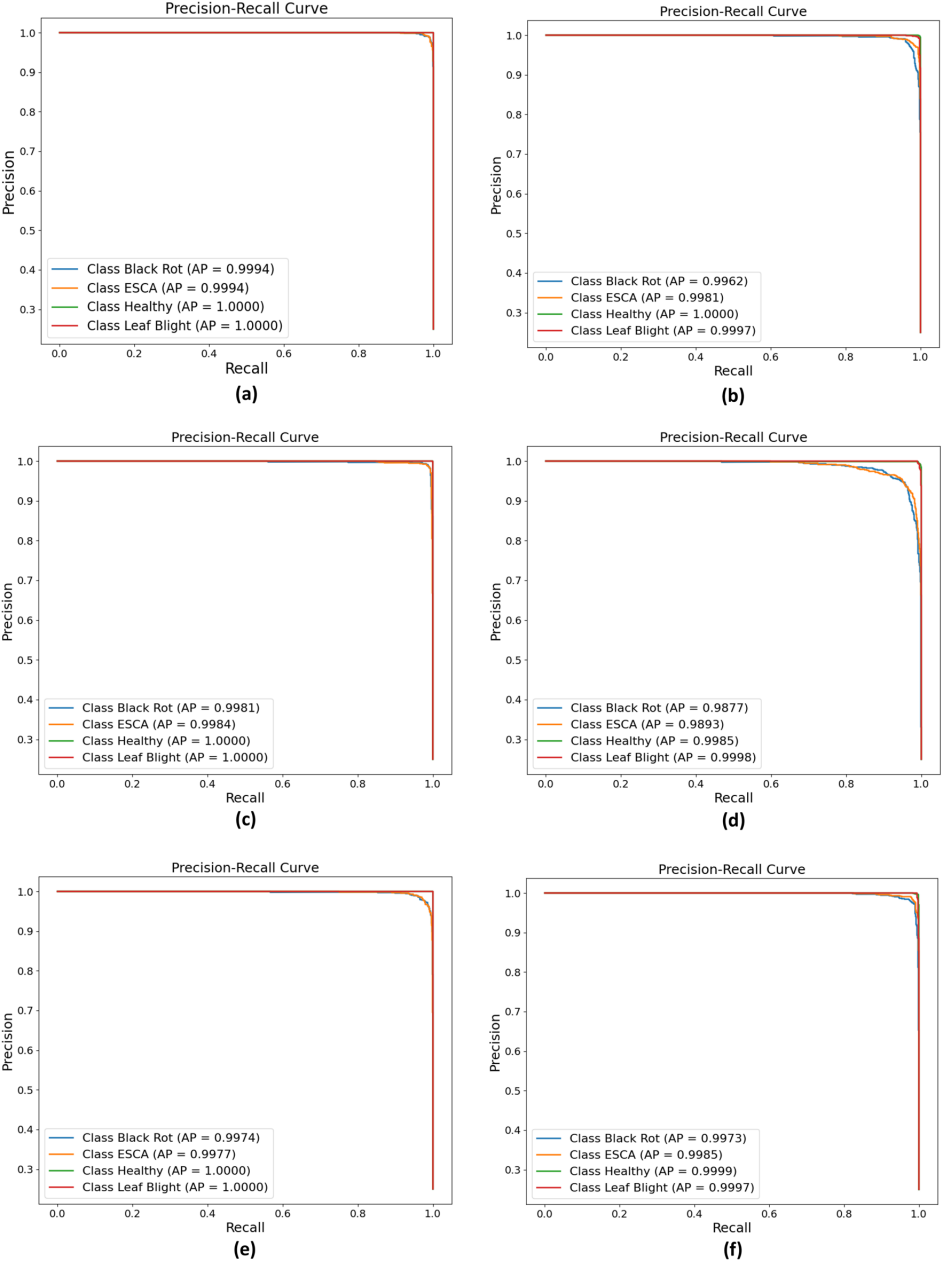
Precision‒recall curves for the (**a**) MobileNetV3Large, (**b**) MobileNetV3Small, (**c**) DenseNet121, (**d**) NASNetMobile, (**e**) EfficientNetV2B1, and (**f**) EfficientNetV2B2 models.

From the obtained ROC curves in Fig. 14, Mobilenetv3Large demonstrated the best correlation between clinical specificity and sensitivity for every potential cutoff, with an AUC for each class very close to one. The trade-off between the true positive rate and the positive predictive value for each predictive model is summarized by precision-recall curves in Fig. 15, where again, MobilenetV3Large has shown better performance than other models, with average precision (AP) values of 99.94% and 100% for the four classes. Based on an analysis of all the performance metrics, MobileNetV3large and DenseNet121 exhibited superior performances. In relation to the model size, performance and parameter count, it may be argued that MobilenetV3Large is more suitable as a lightweight model for execution on edge devices.

**Figure 16:**
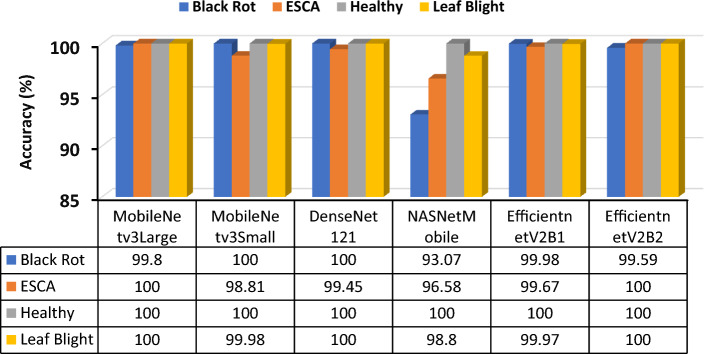
All six model accuracy comparisons for each class prediction.

A comparison bar graph (Fig. 16) was constructed for all six models, where each of the models was used to analyze a random single picture from each of the classes. Based on the model's performance, the models were provided with corresponding confidence scores for each class. The bar chart shows that the proposed MobileNetV3Large model yielded better results than other models in terms of confidence scores during classification.

### Grad-CAM visualization

**Figure 17:**
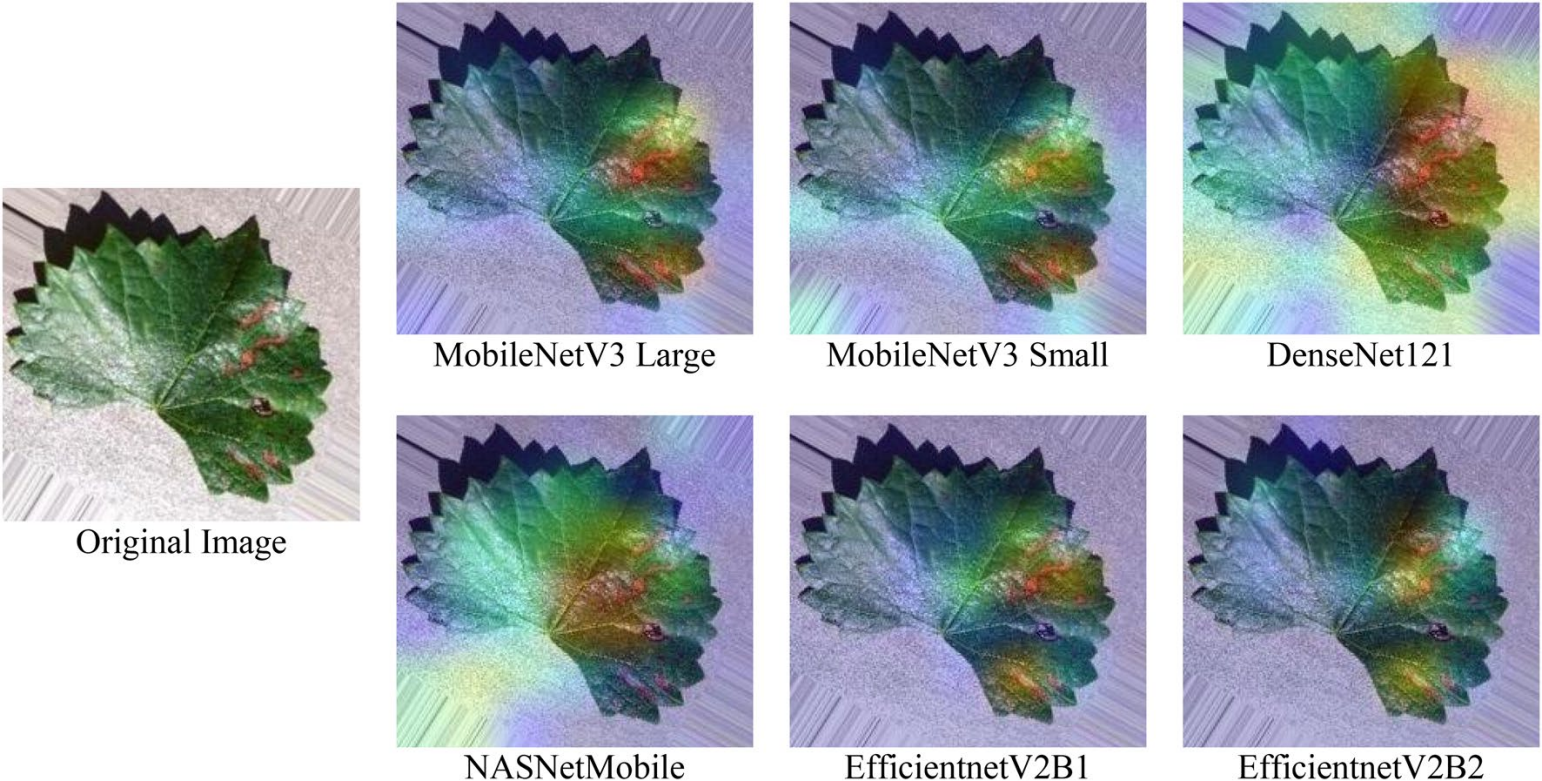
Grad-CAM comparison among all six trained models.

To determine the most important part of the model for neural network prediction, Grad-CAM was applied. A common image was taken on which all six models were used to find their corresponding Grad-CAM visualization, as shown in Fig. 17. Compared with the diseased locations of RGB images, three models, MobileNetV3Large, DenseNet121 and EfficeintNetV2B1, were able to correctly locate those positions well using heatmaps.

### Real-time application of the model in an edge device

The primary aim of this research was to create a lightweight disease classification model that can run on portable edge devices. In addition to the lightweight model, a cost-effective and durable device was also chosen for real-time application.

The NVIDIA Jetson Nano B01 developer kit is a tiny and energy-efficient platform that is well suited for edge and embedded systems, as well as for lightweight CNN models. The device is furnished with a 128-core Maxwell Graphics Processing Unit (GPU) and a quad-core ARM Cortex-A57 Central Processing Unit (CPU), enabling the use of GPU acceleration within the realm of artificial intelligence (AI) and machine learning (ML) applications. The device's minimal power usage and small size make it suitable for incorporation into embedded systems, especially in situations where conserving energy and having limited physical space are crucial factors. The device can function through the utilization of battery power and can be simply housed within small enclosures, making it highly versatile for various edge computing applications^36^.

#### Inference time

**Figure 18:**
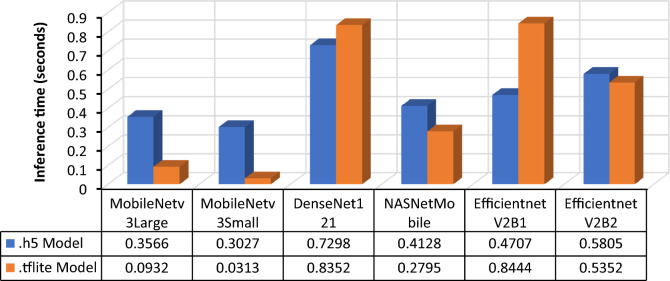
Single image (1 random image) inference time (seconds).

**Figure 19:**
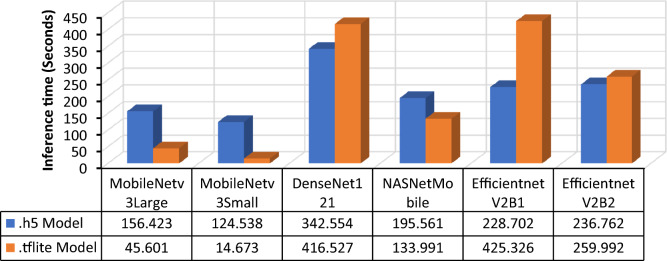
Multiple image (for random and consecutive 500 images) inference time (seconds).

Each of the six models was trained by applying transfer learning, resulting in the acquisition of their respective .h5 models. Subsequently, the models were converted to .tflite format. A specialized memory allocator is used to optimize the execution latency and minimize the computational load. The new file format supported by flat buffers is also being elucidated. TensorFlow Lite is a framework that facilitates the conversion of preexisting models into a more efficient variant encapsulated within the .tflite file format^37^. To enhance the assessment of the six models, both the .h5 and .tflite models were tested, and the resulting prediction performances are presented in Figs. 18 and 19. The results indicated that MobileNetV3Small exhibited the fastest prediction response for both the .h5 and .tflite models, with MobileNetV3Large following closely behind. Anomalies were observed in the performances of the DenseNet121 and EfficientNetV2B1 models, wherein the .tflite model exhibited longer prediction times than did the .h5 model. Despite the slightly faster prediction speed of Mobilenetv3Small, MobilenetV3Large is considered to be the best-performing model due to its superior prediction accuracy.

#### GFLOPS value on an edge device

**Figure 20:**
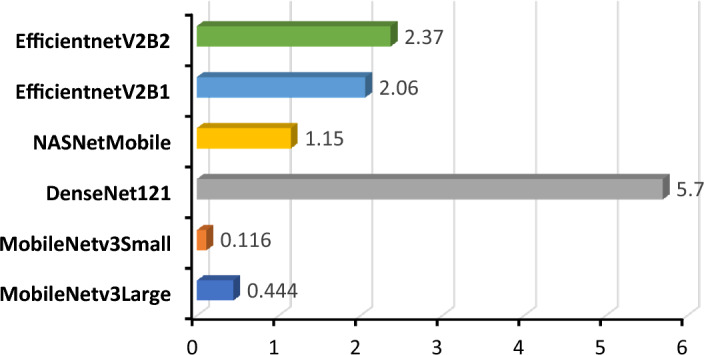
Bar graph of the computational complexity of each model.

Edge devices possess distinct limitations that necessitate the utilization of GFLOPS computations for CNN models on embedded systems with constrained computational capabilities. The measure of GFLOPS holds significant importance to a model for a variety of reasons. The GFLOPS is utilized to assess the computational capacity of a CNN model for the processing capabilities of the device. One can compare GFLOPS data to identify a model that aligns with the capabilities and performance requirements of edge devices. It serves as an indicator of the model's ability to achieve the necessary frame rate or responsiveness for real-time or near-real-time inference applications without excessively burdening the device^38^. The consideration of power consumption also holds significance. Edge devices are frequently operated on batteries or are subject to power limitations. Energy-efficient models such as our MobileNetV3Large (Fig. 20) possess a reduced number of GFLOPS, hence resulting in an extended battery life.

#### Power consumption by the proposed model

**Figure 21:**
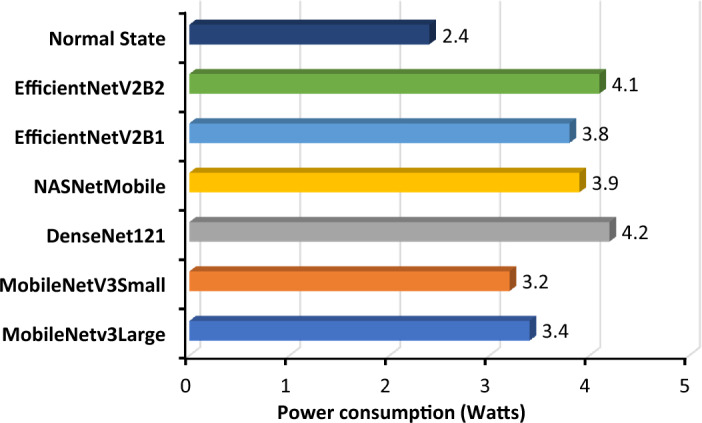
Bar graph of total power consumption while running the models.

Minimizing power consumption is advantageous because it enables devices to operate for extended durations with limited battery power or in remote locations with limited access to electricity sources. Furthermore, this advantage serves to mitigate the generation of excessive heat, thereby preventing potential thermal issues that could impede the overall performance and longevity of the system. A comparison of the power consumption of Jetson Nano during the standby and prediction modes is shown in Fig. 21.

The power consumption of the Jetson Nano was notably low when using MobileNetV3Large, with an average consumption of only 3.4 watts by the device during the prediction of multiple images. The statistics highlight the effectiveness and low resource consumption of the implemented model MobileNetv3Large. This guarantees an extended duration of device functionality, rendering it highly suitable for situations where power resources are limited or when an extended lifespan of the battery is crucial. Consequently, this leads to accelerated inference times, decreased latency, and enhanced real-time performance, which are essential characteristics for applications such as image classification that require prompt decision-making.

### Interface application

**Figure 22:**
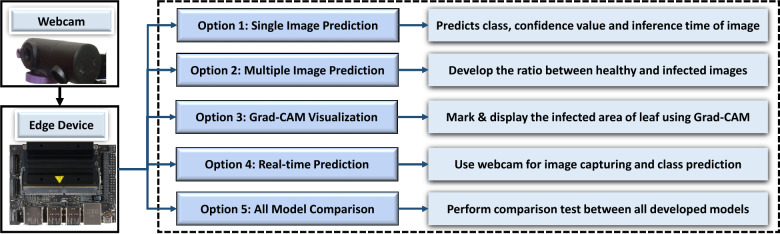
Flow chart for GUI functions.

The use of real-time image prediction facilitates the monitoring of crop growth, identification of optimal harvesting periods, and efficient management of irrigation systems. To perform predictions on our edge device, a custom desktop app was built using Python and PyQt5. Real-time predictions of disease incidence were made available via the Jetson Nano platform. By loading the TensorFlow lite model, the onboard app can predict both real-time and previously saved data as well as use visualization method like Grad-CAM on them. The block diagram for the GUI app operation is shown in Fig. 22.

**Figure 23:**
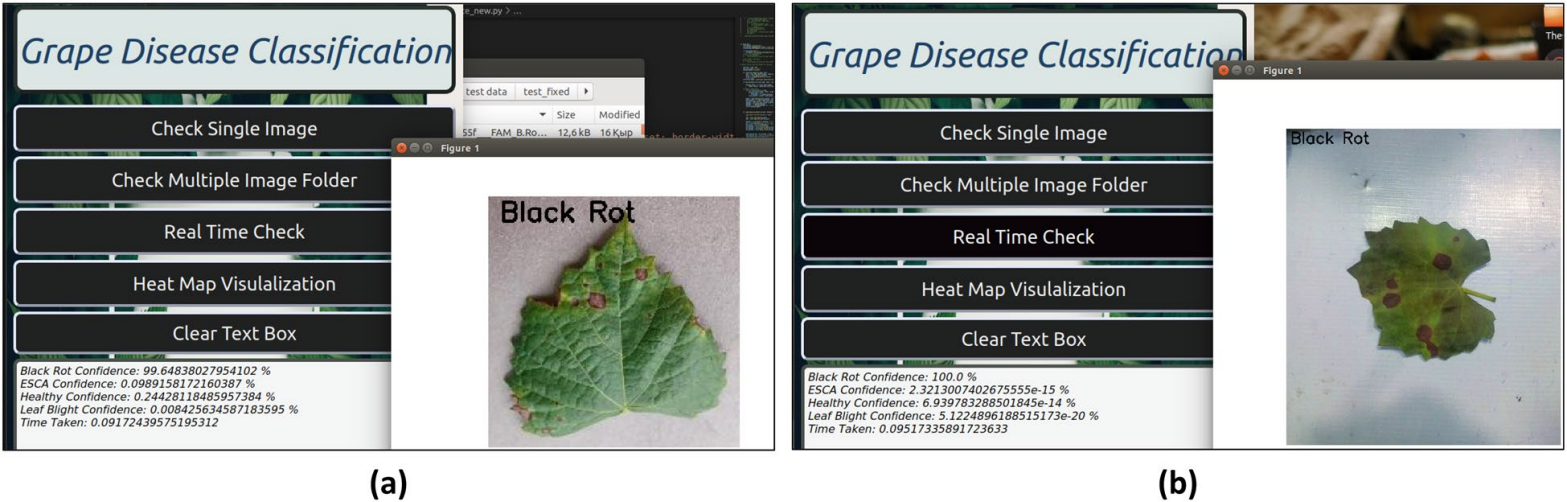
Single-image prediction by GUI. (**a**) Prediction from the saved image. (**b**) Prediction from a real-time image.

PyQt5 is a Python graphics library that offers a comprehensive range of resources for the development of desktop applications featuring graphical user interfaces (GUIs). This software facilitates the creation of interactive and visually captivating applications for diverse platforms, rendering it a widely favored option for developing cross-platform desktop software^39^. The original trained .h5 model was subsequently transformed to the TensorFlow lite model configuration to run on this edge device. The process optimizes models to optimize the efficiency of inference, thereby reducing the demands on memory and computational resources^40^. For user flexibility, both saved image and real-time image classification were made possible using this app interface (Fig. 23). For real-time classification, a Xiaomi Vidlok W91 Webcam was connected to the Jetson Nano board, which allowed to capture live images upon request. The collected frames are then preprocessed, and classification is performed on the GUI app, along with class confidence and time taken for prediction. Before prediction, the sharpness of the collected frame was increased, and its brightness was reduced for better classification. The success of unknown disease classification from this experiment proves the model’s ability to predict unknown data for more practical use.

**Figure 24:**
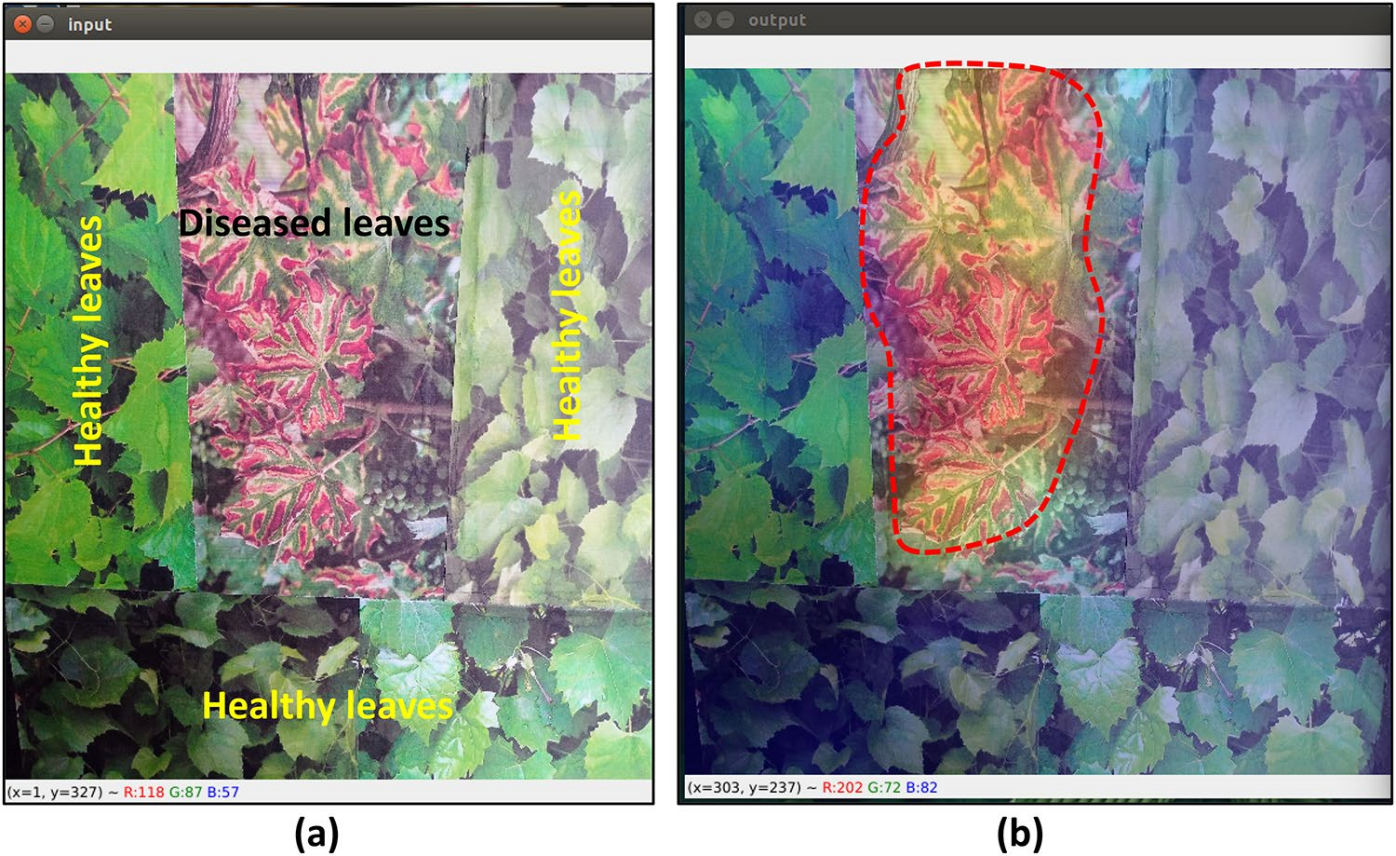
(**a**) input image and (**b**) real-time Grad-CAM visualization in an edge device (correctly identified diseased area is highlighted with dotted line).

The use of the real-time Grad-CAM visualization technique is an effective method for enhancing the visual representation of the impacted region of plants. To assess the efficacy of our customized MobilenetV3Large model in generating heatmaps, a single image depicting a diseased condition was put together with several images portraying healthy conditions, as shown in Fig. 24. By increasing the transparency of the heatmap, the output image presented below demonstrates the model's ability to accurately detect and delineate the affected region only, resulting in a corresponding modification of the color map within that specific area.

### Comparison with previous work

The customized MobileNetV3Large has been compared with existing works in Table 5. Overall, every other study related to grape leaf disease classification claimed that their model was superior to those of previous works. However, upon closer and comprehensive analysis of past works, it was found that almost all of them have some degree of shortcomings. In terms of dataset size, several methods^9,14,18,23^ used a very large number of datasets, which might be convenient. However, upon a detailed look at those datasets, it seems that they might contain duplicate image files, which increases the possibility of overfitting. In the proposed model, the dataset used was cleared of duplicate images using the hash method from both the training and test folders, reducing the chance of making a biased model. The works in^11,13,22^ achieved accuracies of more than 99%, yet the authors did not provide any precision, recall, or F1 score, which raises question on the effectiveness of the model. On the other hand, some works^13,17^ did show these values, but the extremely small size of their dataset proves that these obtained values are not as meaningful as they claimed to be. In this work, along with accuracy, all other important metrics were generated to prove the effectiveness and usability of the proposed method. Two previous works^21,24^ claimed to achieve a higher percentage of accuracy as well as precision, recall, and F1 score. However, upon closer inspection^21^, it was found that their model was severely biased due to duplication of data, and the high values of their evaluation metrics could be due to using the same data for both training and testing purposes.^24^ used a small dataset for six classes, which raises questions about its actual performance in practical usage. According to the authors of developing a lightweight model, the only criteria they presented was model parameter size. However, other factors, such as inference time (Figs. 18 and 19), computational complexity (Fig. 20), and power consumption (Fig. 21), were not discussed.

**Table 5:** Performance comparison with previous works.

References	Method	Accuracy	Precision	Recall	F1-score	Dataset size (images)	Class	Plant
^9^	VGG16	98.40%	–	–	–	62,286	14	Grapes & Tomatoes
^10^	AlexNet, VGG-19, and Inception v3,		–	–	–	3800	4	Grape
^11^	AlexNet	99.23%	–	–	–	4063	3	Grape
^12^	VGG-16	97.87%	–	–	–	17,668	8	Apple & Grape
^13^	UnitedModel(GoogLeNet and ResNet)	99.17%	99.05%	98.88%	98.96%	1619	4	Grape
^14^	Inception structure DICNN	97.22%	97.23%	97.25%	97.40%	**107,366**	7	Grape
^15^	EfficientNetB5	**99.91%**	98.42%	98.31%	–	61,486	**39**	Multicrop
^17^	3D-CNN	95.73%	82.00%	92%	87.00%	1090	2	Grape
^18^	dResNet-18	99.10%	–	–	–	90,000	19	Multicrop
^19^	AlexNet	99.03%	98.90%	98.5%	98.88%	7,222	4	Grape
^20^	AlexNet- ShuffleNetV2 backbone	99.01%	–	–	–	4.062	4	Grape
^21^	MobileNetV3Small	99.00%	**100%**	100.0%	100.0%	58,807	2	Multicrop
^22^	MobileNet-Beta	99.11%	–	92.92%	–	54,306	38	Multicrop
^23^	Faster DR-IACNN(Detection)	81.80%	81.10%	–	–	62,286	4	Grape
^24^	ECA-sNET	99.66%	99.66%	**99.66%**	**99.60%**	6867	6	Grape
Proposed Model	Custom MobileNetV3Large	99.42%	99.42%	99.42%	99.42%	27,122	4	Grape

Significant values are in bold.

One of the most important steps in the proposed research was to apply Grad-CAM not only for model evaluation but also for real-time use in disease localization in large fields. Some previous works^16,24,26^ have provided evidence of Grad-CAM by demonstrating its ability to locate feature pixels of greater importance, but none of these tests include images from real vineyards or real-time visualizations. In this work, real images from webcam were fed directly to the model, and a very accurate heatmap was produced, which demonstrated the suitability of the model for practical application.

### Discussion

In summary, the objective of the present study was to develop a disease classification system specifically designed for grape plants. The system was constructed through the deployment of a lightweight CNN model, wherein the pretrained model employed was MobileNetV3Large. An edge device, namely, the Jetson Nano, was utilized to deploy the system. Furthermore, the system's capabilities were enhanced with the application of real-time Grad-CAM visualization. The motivation for this endeavor is rooted in the pressing need within the agricultural industry to promptly and accurately detect illnesses in grape plants.

The existing techniques utilized for disease identification in grape agriculture have exhibited certain drawbacks in terms of time, power efficiency, and vulnerability to inaccuracies. Therefore, it is imperative to investigate the development of an automated solution that possesses high levels of accuracy and efficiency, as it can greatly revolutionize existing methods of grape cultivation. This technological development facilitates the implementation of the system on the Jetson Nano, an edge device that facilitates immediate, on-site data processing without the requirement of uninterrupted internet connectivity. The adoption of this deployment arrangement signifies a notable progression, as it removes the need for labor-intensive manual checks. In contrast, it offers a swift, efficient, mechanized, and all-encompassing methodology for disease surveillance.

The integration of real-time Grad-CAM visualization boosted the functionality of the system, providing interpretable heatmaps that enhance the precision and dependability of disease identification. The integration successfully established a connection between artificial intelligence and human understanding, hence promoting increased confidence in the system's results.

**Figure 25:**
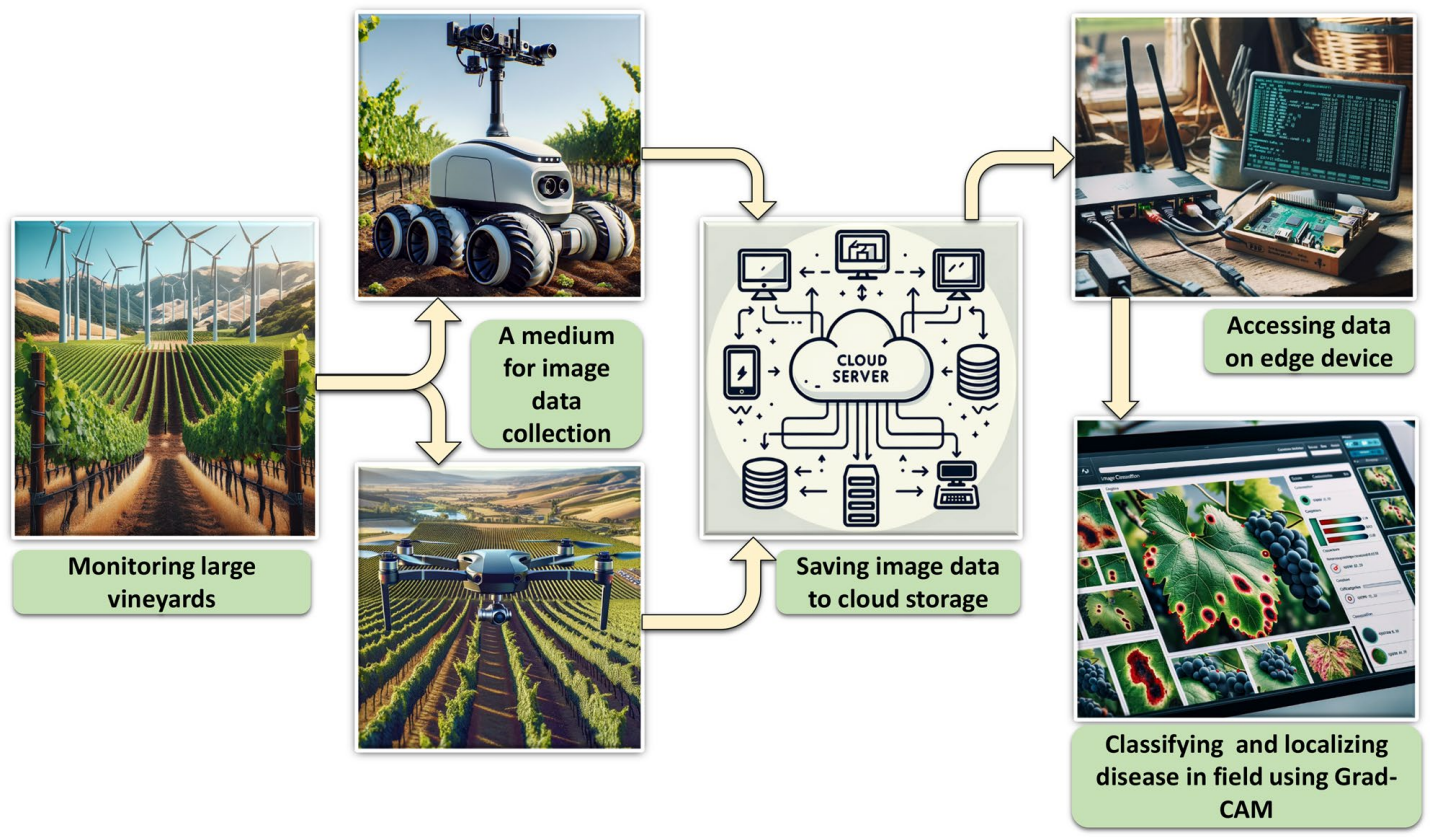
A conceptual schematic diagram of the practical implementation of the proposed grape leaf disease classification system.

The practical implications of this study extend beyond the limitations of the laboratory, offering substantial benefits to the agricultural industry. The overall application concept for this research is presented in Fig. 25. The idea is to focus on a terrestrial rover or an aerial drone fitted with cameras traversing above grapevines, taking visual data from vineyard plants. The data gathered by both the robot and the drone will be sent to a centralized cloud server for storage and distribution. The central computer will identify diseases in grape leaves. Furthermore, Grad-CAM will provide visual information for judgments made by CNNs, emphasizing the specific regions in vineyard photos that are affected by diseases.

It should be noted that the data used for training the model were augmented using general augmentation techniques that sometimes lead to overfitting. Furthermore, both black rot and ESCA diseases look quite similar, which causes the models to slightly mismatch their class during prediction.

## Conclusion

This paper proposed a lightweight customized CNN model that was developed using MobileNetV3Large through transfer learning for running on an edge device. Five other models (MobileNetV3Small, DenseNet121, NASNetMobile, EfficientNetV2B1, and EfficientNetV2B2) were also tested and compared with the proposed MobileNetV3Large model. Based on the results, the optimal model was customized MobilenetV3Large. According to all the experimental results, several conclusions can be drawn.

1. With the same parameter values for all the models during training, the pretrained weights were frozen, and multiple additional dense layers along with dropout layers were included in the final layers of the models. Upon several tests with the values of these layers, dense neurons of 128 and 256 with dropout levels of 0.45 generated the best possible outcome for the proposed classification model. A smaller learning rate and an ideal batch size of 32 also helped in achieving a less unstable training graph.
2. Upon individual inspection of all six models, MobileNetV3Large has the highest training and test accuracies of 99.66% and 99.42%, respectively.
3. The smaller model size, lower prediction time (90 ms), and lower computational resources of the proposed model make it highly suitable for operating on lightweight devices. Additionally, the low power (approximately 1.0 W) used during the prediction shows the potential for practical application.
4. In terms of Grad-CAM visualization, the model was successful at differentiating diseased areas from healthy areas. The heatmap threshold was reduced to a certain portion, making it only focused on greater diseased areas for better visual understanding between the healthy and diseased leaf.
5. The custom user interface for using the model has proven to be user friendly and portable while working outdoors. Implementing this approach on any agriculture-based robot or drone is a notable improvement, enabling more efficient, accurate, and sustainable agricultural operations.

In future work, images from the dataset will be collected from native vineyards with suitable backgrounds. In addition, the dataset will be generated using generative adversarial networks (GANs) to obtain better model performance. This approach aims to introduce diversity into the dataset and improve the effectiveness of anomaly identification. Second, the implementation of an attention module on the existing CNN model helps enhance the model's performance and assists in classifying black rot and ESCA with much precision. Third, the model will be deployed on unmanned aerial vehicles (UAVs) to locate disease-affected regions in vineyards using Grad-CAM for quicker monitoring and accurate localization.

## Data Availability

The datasets used and/or analyzed during the current study will be available from the corresponding author on reasonable request. The dataset is made available at the following link: https://www.kaggle.com/datasets/jawadulkarim117/grape-leaf-disease-4-class.
